# Perspective of Dose and Response for Individualized Physical Exercise and Training Prescription

**DOI:** 10.3390/jfmk5030048

**Published:** 2020-07-14

**Authors:** Thomas Gronwald, Alexander Törpel, Fabian Herold, Henning Budde

**Affiliations:** 1 Faculty of Health Sciences, Department of Performance, Neuroscience, Therapy and Health, MSH Medical School Hamburg, University of Applied Sciences and Medical University, Am Kaiserkai 1, 20457 Hamburg, Germany; 2 German Swimming Federation, Korbacher Straße 93, 34132 Kassel, Germany; toerpel@dsv.de; 3 Research Group Neuroprotection, German Center for Neurodegenerative Diseases (DZNE), Leipziger Str. 44, 39120 Magdeburg, Germany; fabian.herold@st.ovgu.de; 4 Department of Neurology, Medical Faculty, Otto von Guericke University, Leipziger Str. 44, 39120 Magdeburg, Germany; 5 Faculty of Human Sciences, MSH Medical School Hamburg, University of Applied Sciences and Medical University, Am Kaiserkai 1, 20457 Hamburg, Germany; henning.budde@medicalschool-hamburg.de

**Keywords:** dose, acute response, chronic response, internal load, external load, exercise and training prescription, exercise is medicine, personalized medicine

## Abstract

Physical interventions are used to increase physical (sports) performance and considered as effective low-cost strategies in the fields of healthcare, disease or injury prevention, and medical treatment. In general, a considerable amount of evidence buttress the application of physical interventions in various fields as it has been demonstrated to contribute to the maintenance and recovery of physical performance, cognitive function, and overall state of health. To implement physical interventions effectively, it is essential to provide an appropriate exercise and training prescription. Exercise and training prescription are key for “dose” specification and for the individualization (personalizing) of physical exercise and training, precisely adjusted and controlled like medication. Since the physiological response to physical interventions is demonstrably individual and dependent on many influencing factors, individualization is an emerging approach aiming to maximize the efficiency of an intervention by accounting for the interindividual heterogeneity. The present brief viewpoint article aims to distinguish and to redefine between the terms dose and response in order to improve the understanding of practitioners, the methodology of study protocols, and to relate future findings to the actual biological (interindividual) variability of acute and chronic responses.

## 1. Introduction

**Figure 1:**
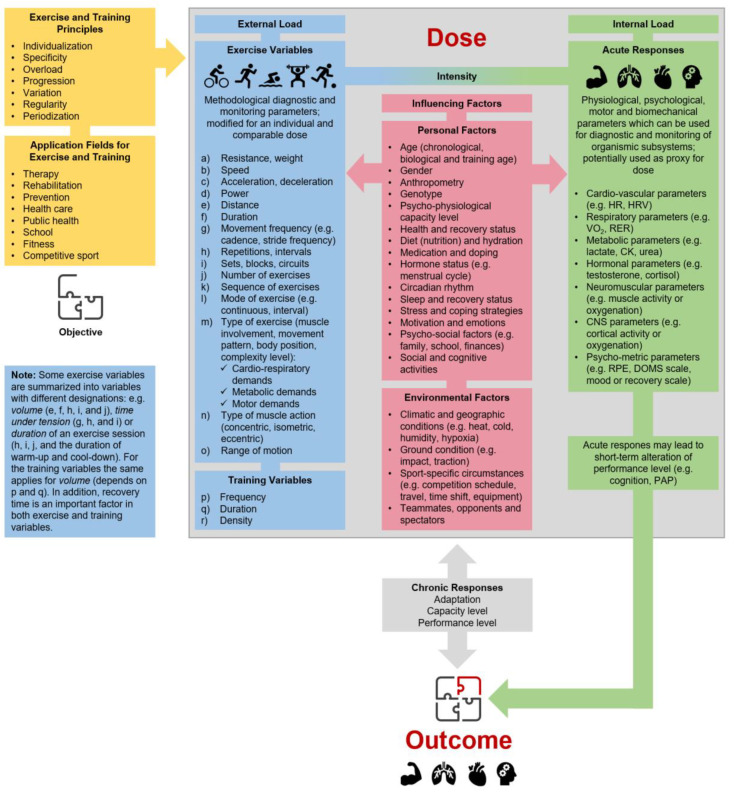
Individual physical exercise and training prescription are based on specific objectives and the respective context in the varying fields of application, as well as principles for the programming and monitoring of physical exercise and training. The dose–outcome relationship depends on a multitude of factors, such as factors of external and internal load and influencing factors. HR: heart rate, HRV: heart rate variability, VO_2_: oxygen uptake, RER: respiratory exchange ratio, CK: creatine kinase, CNS: central nervous system, RPE: rating of perceived exertion, DOMS: rating of delayed onset muscle soreness, PAP: post-activation potentiation.

There is growing evidence that regular physical activity and/or physical exercise (as planned, structured, and purposive forms of physical activity [1,2]) lead to positive effects on physical performance and health in various physiological subsystems (e.g., metabolic, cardiovascular, musculoskeletal, or central nervous system) and the organism as a whole, which emphasizes its use in different fields of application [3,4]. Hence, “physical interventions“, which serve as an umbrella term that covers “physical exercise” (as an acute single bout of physical exercise) and “physical training” (as regularly conducted and multiple bouts of physical exercise [2]), are used and have been proven to be an effective low-cost strategy to recover, maintain or increase physical (sports) performance or the overall health status of an individual in different fields of application (e.g., healthcare, disease and injury prevention, medical treatment). To implement physical interventions effectively in physical (sports) performance enhancement, disease prevention, and medical treatments, it is essential to provide an appropriate exercise and training prescription [5,6]. Such a prescription should consider the fundamental principles of exercise and training prescription (e.g., regularity, overload, progression [7]) and should fully specify external load variables (such as exercise and training variables) and internal load variables (see Figure 1). Furthermore, exercise prescription is key for “dose” or “dosage” (regularly provided dose over a specific period of time) specification and for individualization (personalizing) of physical exercise and training, precisely adjusted and controlled like medication [8,9]. In the following, we are using dose as an umbrella term covering dose and dosage. 

Since the physiological response to physical interventions is demonstrably individual and dependent on many influencing factors, individualization is an emerging approach which aims to maximize the efficiency of an intervention by accounting for the interindividual heterogeneity in athletes, healthy populations and patients [5,10,11,12]. Therefore, it is necessary to evaluate the actual interindividual differences in acute psychophysiological response(s) to the same acute physical exercise and/or adaptations to the same physical training [13,14,15]. To take interindividual heterogeneity into account, a discussion about the classification of “responder”, “non-responder”, “adverse responder”, or “individuals who did not respond” has been emerged [14,16], but a generally accepted agreement on an appropriate classification approach has yet not been reached [15,16,17]. However, the extent of the individual physiological response to physical interventions (sensitivity to respond to the given stimuli) need to be referenced relative to a specific outcome in the variable of interest according to the initial objective. The interindividual responsiveness to physical interventions and, in turn, the interindividual heterogeneity in outcomes are caused by several moderators, including non-modifiable factors (e.g., sex or genotype) and modifiable factors (e.g., nutrition, social or cognitive activities, exercise prescription) [13,14,18,19]. Moreover, it is assumed that low-sensitive responsiveness can be best counteracted by modifying the dose of the physical exercise and/or physical training [20,21]. The latter suggests that the dose of physical interventions per se contributes significantly to the observed interindividual heterogeneity of specific outcomes. In a recent systematic review and meta-analysis, Greenham et al. [22] identified biomarkers of physiological responses associated with altered exercise performance following intensified physical training. The majority of the identified biomarkers demonstrated inconsistent findings, due in part to large interindividual response heterogeneity. The authors recommending that future research should strengthens the focus on individual responses rather than group responses and factors that contribute to the interindividual variability in response. In this regard, the term dose of physical interventions has not yet been clearly defined [23]. The present viewpoint article aims to distinguish between the terms dose and response in order to improve the understanding of practitioners and the methodology of study protocols and to relate future findings to the actual biological (interindividual) variability of acute responses and chronic adaptations.

## 2. Redefining Dose and Response for Individualized Physical Exercise and Training Prescription

An adequate physical exercise and training prescription is a key element in science and practice to characterize the dose of physical interventions. In order to define the dose of a physical intervention, three key components should be considered: (1) external load (defined as the work completed by the individual independent of internal characteristics), (2) influencing factors (all factors that can strengthen or disturb the stimuli of a single bout of exercise and/or training), and (3) internal load (defined as the individual and acute physiological, psychological, motor, and biomechanical responses to the external load and the influencing factors during and/or after the cessation of a single bout of physical exercise) [2,24,25,26,27,28,29,30]. Figure 1 gives an overview of the multitude of factors in the subcategories, without claiming to be complete. In this regard, parameters of external load (e.g., running with a speed of 10km/h or swimming with a pace of 65 s per 100 m) or parameters of internal load (e.g., running with 70% of maximum heart rate) can be used to prescribe and control exercise intensity. Here, the internal load has a key role in physical exercise and training prescription as it represents the crucial impetus for acute and/or chronic changes [18,30,31,32,33,34]. Hence, we propose that dose can be operationalized and monitored using a specific indicator (or set of specific indicators) of internal load as proxy. In this regard, it is mandatory to distinguish with respect to the number of exercise sessions between a single bout of physical exercise (i.e., one session leads to an internal load) and repeated bouts of physical exercise defined as training (i.e., several and consecutive sessions during a defined period lead to repeated bouts of internal loads) [1]. Whereas a single bout of physical exercise leads to distinct acute responses shown by a transient reaction of the organism (beneficial, maintaining, or detrimental depending on the stimuli), repeated bouts of physical exercise ultimately converge into distinct chronic responses (beneficial, maintaining, or detrimental depending on the stimuli). 

With regard to our definition of dose, and given that internal load as acute response is a part of dose, the term “response” in the frequently used phrase “dose–response” should be specified as “chronic response” (effect on a specific outcome parameter, e.g., mitochondrial volume and density) in the meaning of adaptation as a potential result of several and consecutive sessions of physical exercise. To be even more precise and to broaden the understanding of the dose–response relationship, we recommend redefining the phrase “dose–response” as “dose–outcome”, which specifies the link to an acute outcome parameter (in regard to a single bout of physical exercise) or a chronic outcome parameter (in regard to repeated bouts of physical exercise defined as training) according to the respective objective. In this context, dose could be seen as an independent variable or a set of independent variables which we assume to be involved in biological processes in general and in a complex response matrix and signal transduction [35], specifically leading to a distinct “outcome” (dependent variable). However, according to the definitions, internal load as proxy of the dose could be controlled by modifying the external load in consideration of exercise and training principles (e.g., periodization for the planned systematic and structural variation of a training program over time with an adequate ratio of load and recovery periods) and influencing factors such as the actual state of the psychophysiological capacity level (including level of performance).

## 3. Implications and Areas for Future Research

Valid indicators that represent the most appropriate proxies of dose for prescribing physical interventions are highly specific and more research is needed to identify them (with regard to the context and/or specific acute or chronic responses) [18]. In this regard, current concepts discuss promising internal load parameters (e.g., brain-derived parameters, hormones) to prescribe physical exercise, in addition to traditional measures like heart rate, blood lactate concentration, or rating of perceived exertion [36]. Nevertheless, there is a good, at least theoretical, rationale in support of the individualization of exercise and training prescription by providing a distinct (comparable and standardizable) dose across individuals to elicit the desired psychophysiological responses, which would in turn allow for a better comparison of outcomes across different individuals [2,37,38]. Therefore, existing recommendations endorse the adequate prescription of single exercise sessions and/or training with the specification of parameters of external load and markers of internal load in science and practice [31,32,38,39]. Furthermore, regarding controlled trials of physical interventions and difficulties for blinding participants, it is advisable to include a sham condition in order to avoid potential biases for at least some of a multitude of influencing factors regarding the positive effects of physical activity and physical exercise. A sham intervention should be designed very specifically and should aim to closely replicate virtually all of the elements of a physical exercise condition, regarding variables of physical exercise and physical training (e.g., setting and equipment, socialization, supervision, care, motivation and counselling, outcome expectations, modality and type of exercise, volume, duration, movement frequency, training frequency and density, e.g., [18,40,41]), with the exception of important (hypothesized) prescriptive elements leading to targeted outcomes (e.g., exercise intensity, progression over time). Promising methodological approaches already exist for this purpose [42]. The importance of controlling for social support when designing interventions, which points out the need for adequate sham intervention, has also been highlighted by different authors [43,44]. This approach will further ensure high quality standards for the evaluation of exercise and training prescription and the dose effects of physical interventions.

## 4. Conclusions

In essence, this brief opinion provides a new and clearer definition of the terms dose and response in the context of exercise and training prescription. We propose that the dose of physical exercise and/or physical training should be operationalized by a specific marker (or specific markers) of internal load. Modifying the exercise prescription by carefully adjusting the external load, a comparable dose can be achieved across individuals, discovering the “real” interindividual heterogeneity regarding acute and chronic responses to physical interventions. We strongly encourage researcher to investigate whether exercise and training prescription that induces a comparable dose may reduce the interindividual heterogeneity considering specific (targeted) outcome variables [45].
